# Human Monocytes Are Suitable Carriers for the Delivery of Oncolytic Herpes Simplex Virus Type 1 In Vitro and in a Chicken Embryo Chorioallantoic Membrane Model of Cancer

**DOI:** 10.3390/ijms24119255

**Published:** 2023-05-25

**Authors:** Alberto Reale, Lea Krutzke, Massimiliano Cadamuro, Adriana Vitiello, Jens von Einem, Stefan Kochanek, Giorgio Palù, Cristina Parolin, Arianna Calistri

**Affiliations:** 1 Department of Molecular Medicine, University of Padua, 35121 Padua, Italy; massimiliano.cadamuro@unipd.it (M.C.); adriana.vitiello@unipd.it (A.V.); giorgio.palu@unipd.it (G.P.); cristina.parolin@unipd.it (C.P.); 2 Department of Gene Therapy, Ulm University Medical Center, 89081 Ulm, Germany; lea.krutzke@uni-ulm.de (L.K.); stefan.kochanek@uni-ulm.de (S.K.); 3 Institute of Virology, Ulm University Medical Center, 89081 Ulm, Germany; jens.von-einem@uni-ulm.de

**Keywords:** oncolytic virus, virotherapy, HSV-1, monocytes, carrier cells, CAM model

## Abstract

Oncolytic viruses (OVs) are promising therapeutics for tumors with a poor prognosis. An OV based on herpes simplex virus type 1 (oHSV-1), talimogene laherparepvec (T-VEC), has been recently approved by the Food and Drug Administration (FDA) and by the European Medicines Agency (EMA) for the treatment of unresectable melanoma. T-VEC, like most OVs, is administered via intratumoral injection, underlining the unresolved problem of the systemic delivery of the oncolytic agent for the treatment of metastases and deep-seated tumors. To address this drawback, cells with a tropism for tumors can be loaded ex vivo with OVs and used as carriers for systemic oncolytic virotherapy. Here, we evaluated human monocytes as carrier cells for a prototype oHSV-1 with a similar genetic backbone as T-VEC. Many tumors specifically recruit monocytes from the bloodstream, and autologous monocytes can be obtained from peripheral blood. We demonstrate here that oHSV-1-loaded primary human monocytes migrated in vitro towards epithelial cancer cells of different origin. Moreover, human monocytic leukemia cells selectively delivered oHSV-1 to human head-and-neck xenograft tumors grown on the chorioallantoic membrane (CAM) of fertilized chicken eggs after intravascular injection. Thus, our work shows that monocytes are promising carriers for the delivery of oHSV-1s in vivo, deserving further investigation in animal models.

## 1. Introduction

Oncolytic viruses (OVs), by definition, are able to selectively kill cancer cells, and thus can be used as antitumoral therapeutics [1]. The main mechanism underlying this selectivity is the impaired function of intrinsic cellular antiviral pathways [2], which facilitates the replication of attenuated viruses in malignant cells. Furthermore, OVs are also considered to be a form of immunotherapy, eliciting an antitumoral immune response [2]. Finally, they can also be armed to express therapeutic genes, thus improving their efficacy [3]. Most OVs are based on attenuated human pathogens or wild-type viruses which normally infect other animals and do not cause disease in humans [4,5,6]. The attenuation of human viruses aims at reducing their capability to downregulate antiviral pathways in healthy cells. In the case of oncolytic herpes simplex virus type 1 (oHSV-1), both copies of the γ34.5 gene are usually deleted [7]. The protein encoded by γ34.5, infected cell protein (ICP)34.5, antagonizes different cellular antiviral mechanisms, including protein kinase triggered by double-stranded RNA (PKR)-mediated protein synthesis shutoff [8], the activation of TRAF family member associated NFKB activator (TANK)-binding kinase 1 (TBK1) [9] and Beclin-1-mediated autophagy [10]. Another gene which has been deleted in the oHSV-1 genome is US12, whose product, ICP47, reduces MHC-I-mediated antigen presentation in infected cells [11].

Talimogene laherparepvec, also known as T-VEC, is an oHSV-1 bearing both of the above mentioned mutations. T-VEC is additionally armed with two copies of the immunostimulatory human cytokine granulocyte–monocyte colony stimulating factor (hGM-CSF) [12]. T-VEC was the first OV to be approved by the Food and Drug Administration (FDA) and by the European Medicines Agency (EMA) for the intralesional treatment of unresectable melanoma [13], demonstrating that OVs represent a promising and versatile weapon for the treatment of many tumors that, unfortunately, still have a very poor prognosis [14]. Most OVs, including T-VEC, are designed for intratumoral injection, since intravenously administered viral particles become rapidly sequestered by off-target cell infection and by the immune system [6,15]. Indeed, systemically administered OVs are challenged by different antiviral immune responses, e.g., cytokines and antibodies, that affect their therapeutic efficacy. This drawback is of particular concern for OVs based on pathogens characterized by a high seroprevalence in the human population, such as HSV-1 [16]. Furthermore, systemic administration is associated with a scarce tumor targeting, the viral uptake/dilution of the virus by off-target organs (especially liver and spleen) and resulting toxicity.

On the other hand, the advantages of systemic administration would be undeniable, particularly when targeting metastases, micrometastases and deep-seated tumors. A strategy to address the obstacle of virus sequestration is the use of so-called carrier cells, which are loaded ex vivo with OVs and are then injected systemically. Such cells should be able to efficiently migrate towards tumor sites and should preferably be autologous. Mesenchymal stem cells (MSCs) have been investigated as carrier cells in preclinical models and in some clinical trials [17]. In addition, other cell types, in particular, cells belonging to the immune system, have also been taken into account [18]. Among different candidate carrier cells for oHSV-1, circulating CD14^+^ monocytes are especially interesting, due to several favorable features: (i) autologous monocytes can be easily recovered in large numbers from the peripheral blood of patients, since they constitute roughly 10% of circulating leukocytes [19] and (ii) they are, at least in part, the precursors of tumor associated macrophages (TAMs) in different malignancies [20], including breast cancer [21,22], colorectal and lung carcinoma [23], glioblastoma [24], and head-and-neck cancer [25]. Therefore, circulating monocytes are actively recruited by many solid tumors through different signaling axes, such as CCL2/CCR2 [26] or CCL5/CCR5 [27]. Thus (iii), considering the vital role that TAMs play in supporting the survival of cancer cells [28], it seems unlikely that tumors may become resistant to a monocyte-conveyed treatment by excluding these cells from the tumor microenvironment (TME), as it happens in the case of T lymphocytes [29].

While few studies have tested monocytes or macrophages as carrier cells for OVs, in association with oncolytic adenoviruses [30,31] and measles virus [32], these cells have never been evaluated as carriers for oHSV-1. Therefore, we investigated whether an enhanced green fluorescent protein (EGFP)-expressing oHSV-1 bearing deletions of the γ34.5 and US12 genes (EGFP-oHSV-1) could infect a human monocytic cell line (THP-1) and primary CD14^+^ monocytes from healthy blood donors and assessed whether EGFP-oHSV-1-infected monocytes could migrate towards cancer cells of different origin in vitro. Finally, we analyzed the tumor tropism of EGFP-oHSV-1-infected THP-1 cells in a more complex experimental setting that includes a complete circulatory system. In particular, we adopted human head-and-neck (larynx) carcinoma xenograft tumors (UM-SCC-11B cells) grown on the chorioallantoic membrane (CAM) of fertilized chicken eggs that are naturally immunocompromised at an early developmental stage [33,34,35,36]. Tumor targeting efficiencies and the biodistribution profiles of EGFP-oHSV-1-infected THP-1 were analyzed. Our results show that: (i) EGFP-oHSV-1 enters human THP-1 and primary monocytes; (ii) productive replication in primary monocytes is significantly boosted when cells are cultured in cancer cell-conditioned media; (iii) infected primary monocytes are able to migrate towards the supernatants of breast and head-and-neck cancer cells in vitro and to transmit the infection to cancer cells in coculture assays; and (iv) in a CAM model, EGFP-oHSV-1-infected THP-1 cells migrate selectively towards UM-SCC-11B tumors after intravascular injection, with very low or no off-target infiltration in the embryo organs.

Overall, our findings suggest that monocytes have different features that make them promising carrier cells for the systemic delivery of oHSV-1.

## 2. Results

### 2.1. THP-1 Cells Can Be Infected by EGFP-oHSV-1

To assess the ability of monocytic cells to become infected with oncolytic herpes simplex virus type 1 (oHSV-1), THP-1 cells were infected with an oHSV-1 expressing the green fluorescence reporter protein (EGFP-oHSV-1) with a multiplicity of infection (MOI) of 1, 3 and 5 plaque forming unit (PFU)/cell.

**Figure 1:**
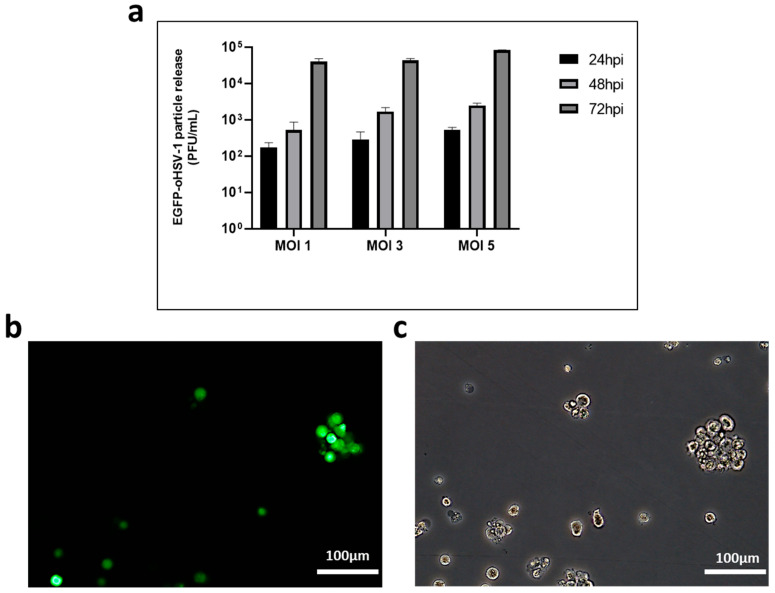
Monocytic THP-1 cells are susceptible to EGFP-oHSV-1 infection and support viral replication. (**a**) Human THP-1 cells (1 × 10^5^) were infected with EGFP-oHSV-1 at the MOI of 1, 3 and 5 PFU/cell, respectively. Cell supernatants were harvested at indicated times post-infection and analyzed for infectious viral particles using plaque assay on Vero cells. Shown are mean virus titers and standard deviation from three replicates, where the *y* axis is in logarithmic scale; (**b**,**c**) Representative images of EGFP-oHSV-1-infected THP-1 cells (MOI 1 PFU/cell) at 72 h post-infection (hpi) taken with a magnification of 20× by fluorescence microscopy (**b**) and by brightfield microscopy (**c**) are shown.

The replication kinetics of EGFP-oHSV-1 were similar at the three different MOIs used for infection, reaching titers of 4 × 10^5^, 4.3 × 10^5^ and 8.2 × 10^5^ PFU/mL, respectively, at 72 h post-infection (hpi) (Figure 1a). Fluorescence microscopy analysis showed that roughly 30% of the THP-1 cells expressed the reporter protein EGFP at the same time post-infection (Figure 1b). Overall, these findings indicate that THP-1 cells are susceptible and permissive to EGFP-oHSV-1 infection.

### 2.2. EGFP-oHSV-1 Infection Is Transmitted by Monocytes to MDA-MB-231 and UM-SCC-11B Cells in a Coculture Assay

**Figure 2:**
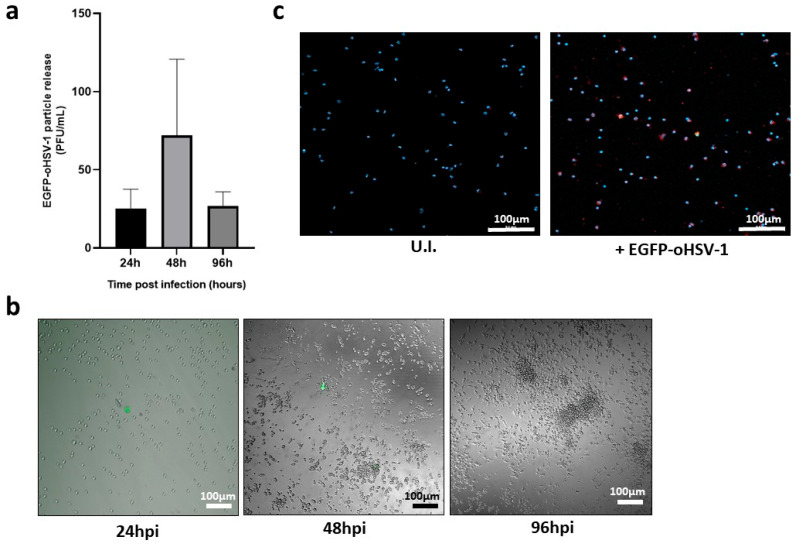
Primary human monocytes are susceptible to oHSV-1 infection although they do not support viral replication. Primary human monocytes (10^5^ cells in 500 µL) were infected with EGFP-oHSV-1 at a MOI of 5 PFU/cell. (**a**) Cell supernatants were harvested at 24, 48 and 96 hpi and infectious viral particles were quantified through plaque titration assay. Experiments were repeated three times and the mean values are reported in the graph, along with the standard deviations; (**b**) 10× fluorescence/brightfield microscopy pictures of EGFP-oHSV-1-infected primary monocytes were taken at indicated times post-infection. Representative images are displayed. Green fluorescence indicates EGFP expression. Scale bars are shown; (**c**) 10× fluorescence/brightfield microscopy pictures of uninfected (U.I.) or EGFP-oHSV-1-infected primary monocytes (24 hpi). Cells were fixed and permeabilized in 100% methanol, then stained with a primary mouse anti-ICP4 antibody (Abcam, Cambridge, UK) and a secondary TexasRed-conjugated goat anti-mouse antibody (Invitrogen, Waltham, MA, USA). Nuclei were marked with Draq5 (Invitrogen). Scale bars are shown.

Having shown that THP-1 cells can be infected with EGFP-oHSV-1, we moved to primary human monocytes isolated from blood of healthy donors. Monocytes were isolated from the buffy coat through Ficoll Paque Plus (Merck) gradient purification and adhesion, resulting in a purity of roughly 80%, as determined by CD14 staining (Figure S1). Purified monocytes were infected with EGFP-oHSV-1 at the MOI of 5 PFU/cell for 1 h. Afterwards, cells were carefully washed to remove not adsorbed infectious virions. The absence of infectious viral particles in the last wash was confirmed via titration on Vero cells. A limited amount of infectious viral particles was measured in the cell supernatant at all analyzed time points post-infection (Figure 2a) with a peak at 48 hpi. The latter titer corresponded to roughly 0.005 virions per cell. In agreement, few EGFP-expressing cells were observed at 24 hpi and 48 hpi and none at 96 hpi (Figure 2b). Moreover, cell viability was not affected compared to uninfected monocytes up to 5 days post-infection. Thus, to confirm cell susceptibility to viral infection, the expression of an HSV-1 immediate early (IE) gene product, the infected cell protein 4 (ICP4), was investigated. Indeed, the expression of IE proteins can also be found in abortively infected cells, thus representing useful markers of cell susceptibility even when the viral replication cycle was not completed and no infectious viral particles were produced [37,38]. At 24 hpi ICP4 was clearly detectable in monocytes exposed to EGFP-oHSV-1 (Figure 2c), supporting viral infection. Importantly, monocytes were negative for ICP4 when analyzed after 1 h of viral adsorption (Figure S2), indicating that the ICP4 signal found at 24 hpi was mostly due to de novo protein synthesis upon viral entry into cells.

**Figure 3:**
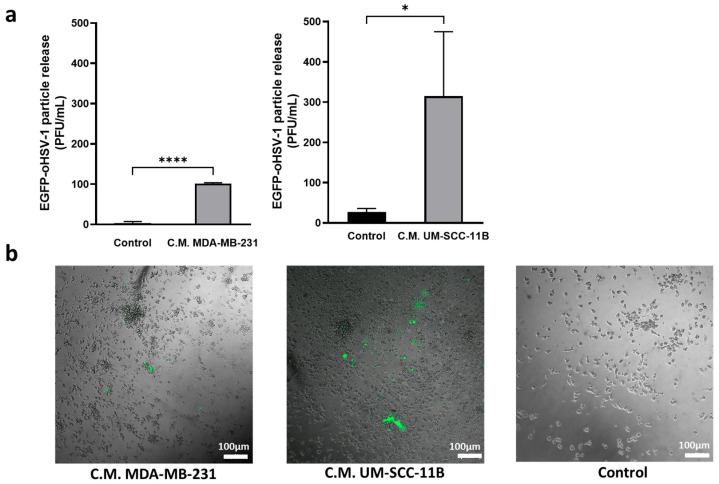
Productive infection with oHSV-1 in primary monocytes is boosted by cancer cell-conditioned medium. Primary monocytes were infected with EGFP-oHSV-1 (MOI 5 PFU/cell) for 1 h and then cultured in RPMI with 10% v/v of fetal calf serum (FCS) (control) or RPMI 10% FCS conditioned by either MDA-MB-231 (C.M. MDA-MB-231) or UM-SCC-11B cells (C.M. UM-SCC-11B). (**a**) Cell supernatants were harvested at 3 (MDA-MB-231) or 5 (UM-SCC-11B) days post-infection and viral particles titrated by plaque assay on Vero cells. Experiments were repeated three times and the mean values are reported in the graph, along with the standard deviations. Asterisks highlight statistically significant differences (**** *p* < 0.0001 and * *p* < 0.05, respectively, Student’s *t*-test); (**b**) Fluorescence microscopy of primary human monocytes infected with EGFP-oHSV-1 (MOI 5 PFU/cell) and then cultured for 5 days in RPMI 10% FCS conditioned by MDA-MB-231 cells (C.M. MDA-MB-231), RPMI 10% FCS conditioned by UM-SCC-11B cells (C.M UM-SCC-11B) or RPMI 10% FCS (Control). Green fluorescence indicates EGFP expression. Images from panel (**b**) are representative of multiple 10× fields. Scale bars are displayed.

When circulating monocytes enter the tumor microenvironment (TME), they undergo differentiation towards tumor associated macrophages (TAMs) [39]. Furthermore, the differentiation and polarization of myeloid cells can affect their susceptibility to HSV-1 infection [40]. Therefore, primary monocytes were infected with EGFP-oHSV-1 (MOI 5 PFU/cell), as described above, and cultured in medium conditioned using MDA-MB-231 breast cancer cells or UM-SCC-11B laryngeal cancer cells. Unconditioned medium was adopted as a control. Cell supernatants were collected at different time points post-infection and released viral particles were titrated on Vero cells. Results show that viral replication was significantly increased compared to the control when monocytes were maintained in cancer cell-conditioned medium (Figure 3a). This finding was also confirmed via fluorescence microscopy performed 5 days post-infection. Indeed, EGFP-positive monocytes could still be detected when monocytes were maintained in medium conditioned with either MDA-MB-231 or UM-SCC-11B cancer cells, whereas no signal was found in the control cells (Figure 3b).

**Figure 4:**
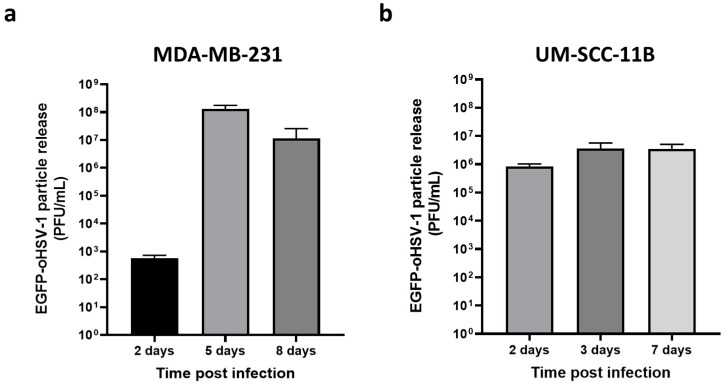
Cancer cells cocultured with EGFP-oHSV-1-loaded primary monocytes are efficiently infected and support viral replication. Primary human monocytes were infected with EGFP-oHSV-1 (MOI of 5 PFU/cells) and washed 3× with PBS to remove extracellular virions. One hour later, infected monocytes were cultured at a 1:1 ratio with confluent (**a**) MDA-MB-231 or (**b**) UM-SCC-11B cells. Supernatants were harvested at the indicated time points and titrated via plaque assay on Vero cells. Obtained values are reported in graphs where the *y* axis is in logarithmic scale. Experiments were repeated three times and mean values are reported along with standard deviations.

Having demonstrated that monocytes can be loaded with EGFP-oHSV-1 and that viral replication was favored by cancer cell-conditioned medium, we investigated whether infected monocytes could transmit EGFP-oHSV-1 infection to MDA-MB-231 and UM-SC-11B cells. To this end, a coculture assay was set up. Briefly, primary human monocytes were incubated for 1 h with EGFP-oHSV-1 at a MOI of 5 PFU/cell and washed three times with PBS. The last wash was controlled for the absence of infectious viral particles using plaque assay on Vero cells. Monocytes exposed to EGFP-oHSV-1 were cultured together with MDA-MB-231 or UM-SCC-11B cells at a 1:1 ratio. At different time points post-coculture, cell supernatants were harvested and the presence of infectious viral particles was analyzed via plaque titration assay. Viral titers increased up to 7 days post-infection for both the MDA-MB-231 (Figure 4a) and the UM-SCC-11B (Figure 4b) cocultures. Importantly, viral yields in supernatants in MDA-MB-231 cocultures as well as in UM-SCC-11 cocultures were significantly higher (*p* < 0.001) than those obtained in previous experiments for the infected monocytes maintained either in non-conditioned medium (Figure 2a) or in cancer cell-conditioned medium (Figure 3a), suggesting a contribution of cancer cells in supporting viral replication.

**Figure 5:**
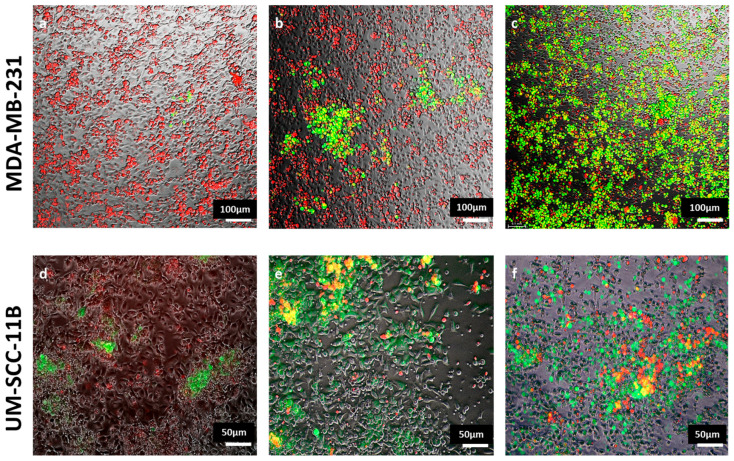
Monocytes loaded with oHSV-1 efficiently transmit viral infection to cancer cells. EGFP-oHSV-1-infected primary monocytes (MOI of 5 PFU/cell) were marked with the vital CellTracker™ Red CMTPX dye (Invitrogen) and cocultured at a 1:1 ratio with MDA-MB-231 or UM-SCC-11B cells. The 10× pictures of monocytes-MDA-MB-231 cocultures were acquired 1 day (**a**), 2 days (**b**) or 6 days (**c**) post-infection. The 10× pictures of monocytes-UM-SCC-11B cocultures were acquired 1 day (**d**), 2 days (**e**) and 3 days (**f**) post-infection. Images from panels are representative of multiple 10× fields. Green fluorescence indicates EGFP expression, red fluorescence indicates primary monocytes.

To further corroborate this conclusion and confirm the infection of cancer cells, monocytes were marked with a vital fluorescent red dye before being cocultured with MDA-MB-231 cells, as described above. Cells were followed by live fluorescence microscopy showing the infection of both monocytes and cancer cells by EGFP-oHSV-1. As displayed in Figure 5 (upper panels), the number of green only cells, which are EGFP-positive cancer cells, increased over time, accompanied by a cytopathic effect (cellular rounding and detachment), particularly at later time points. Notably, the number of yellow cells, indicative of infected monocytes, also increased over time. This finding supports the data reported in Figure 3, showing that viral replication is boosted in monocytes, in an environment conditioned by cancer cells. Similar results were also obtained in the case of the UM-SCC-11B coculture (Figure 5, lower panels).

### 2.3. EGFP-oHSV-1-Infected Monocytes Migrate towards Supernatants of Cancer Cells

**Figure 6:**
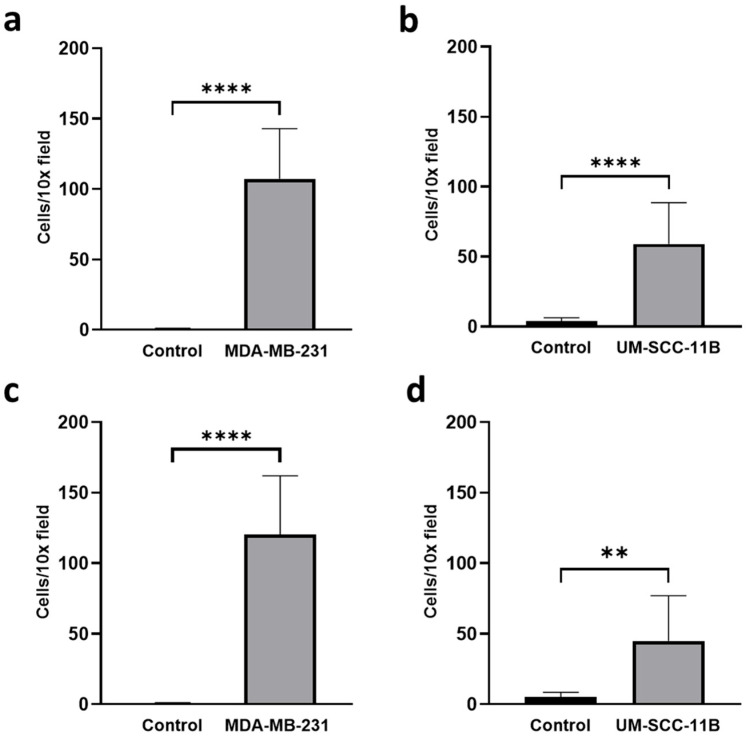
Primary human monocytes infected with EGFP-oHSV-1 migrate towards serum-free medium conditioned by cancer cells as efficiently as mock-infected controls. Mock-infected (**a**,**b**) or EGFP-oHSV-1 (MOI = 5 PFU/cell)-infected (**c**,**d**) primary monocytes (10^5^ cells) were marked with CellTracker™ Red CMTPX Dye (Invitrogen) and allowed to migrate towards serum-free medium (OptiMEM, Gibco, Waltham, MA, USA) or serum-free medium conditioned by MDA-MB-231 (**left**) or UM-SCC-11B (**right**) cells through a 5.0 µm-pore filter for 3 h at 37 °C in a 5% CO_2_ atmosphere. Fluorescent cells were counted from at least three independent 10× microscopic fields in the lower chamber. Error bars indicate standard deviation. The difference is statistically significant (**** *p* < 0.0001 and ** *p* < 0.01, respectively, Student’s *t*-test).

Once we assessed that monocytes were able to efficiently transmit oHSV-1 to cancer cells in co-culture experiments, we investigated whether infected monocytes were able to migrate toward an environment conditioned by cancer cells. To this end, we adopted a Boyden chamber migration assay by employing inserts with 5.0 µm pores. In this assay, monocytes were first allowed to migrate for 3 h through the inserts towards control serum-free medium as well as towards serum-free medium conditioned by MDA-MB-231 or by UM-SCC-11B cells. Cells were marked with a vital red dye and counted in three different 10× microscopy fields. As expected, the average number of primary human monocytes that migrated towards MDA-MB-231 (Figure 6a) and UM-SCC-11B (Figure 6b) conditioned medium was significantly higher than the average number of infected monocytes that migrated towards control medium. Next, the same migration assay was repeated by adopting primary monocytes infected with EGFP-oHSV-1 at the MOI of 5 PFU/cell for 1 h. Infected cells were assayed for their migration ability, as described for mock-infected monocytes. Data show that the migration of infected monocytes towards MDA-MB-231 (Figure 6c) and UM-SCC-11B (Figure 6d) conditioned medium is not any different from what is seen with uninfected monocytes (Figure 6a and Figure 6b, respectively).

### 2.4. EGFP-oHSV-1-Infected THP-1 Cells Migrate Selectively towards UM-SCC-11B Tumors in a CAM Model of Head-and-Neck Cancer

**Figure 7:**
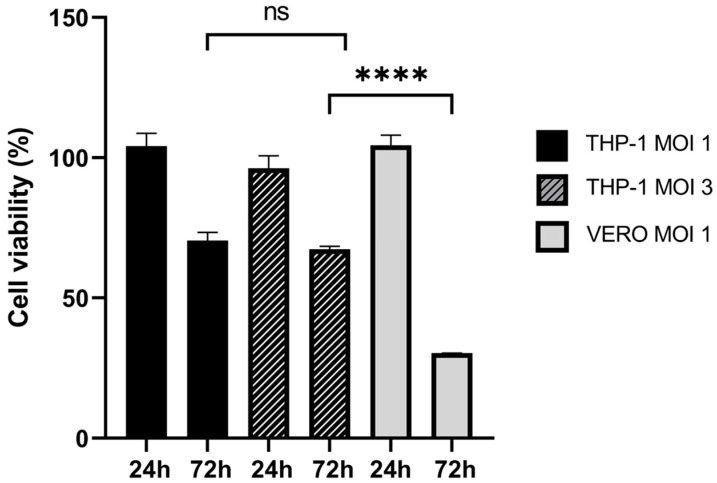
Most THP-1 cells remain viable up to 72 h after oHSV-1 infection. THP-1 cells were infected with EGFP-oHSV-1 at the MOI of 1 or 3 and their viability activity was measured at 24 and 72 hpi using the Cell Proliferation Kit I (Roche Diagnostics, Monza, Italy). Values reported on the *y* axis are expressed in %, calculated from the ratio of absorbance (580 nm) obtained from infected cells and uninfected cells cultivated with the same conditions. For comparison, fully susceptible Vero cells were infected with the same virus at the MOI of 1 PFU/cell and their metabolic activity was evaluated at 24 and 72 hpi. **** highlight statistically significant difference (*p* < 0.0001, Student’s *t*-test); ns stands for no statistically significant difference (*p* > 0.05, Student’s *t* test).

Having demonstrated in vitro that monocytes infected with oHSV-EGFP are still able to migrate towards conditioned cancer medium, we moved to a more complex experimental setting to assay the ability of the carrier cells under evaluation to reach tumor beds in vivo. In this series of experiments, we selected THP-1 cells as carriers since these cells do not need a purification step and can be rapidly amplified. First of all, we analyzed THP-1 viability over 3 days upon infection with EGFP-oHSV-1 at a MOI of 1 or 3 PFU/cell compared to uninfected cells. Although a reduction (30% and 33%, respectively) was found at 72 hpi, this was significantly lower than the one observed at the same time point when a highly susceptible cell line (Vero cells) was infected (Figure 7). Interestingly, no statistically significant difference between the survival of cells infected at MOI of 1 and MOI of 3 at 72 hpi was observed (Figure 7). Therefore, to maximize viral delivery to the tumor, the higher MOI of 3 PFU/cell was selected for the following experiments.

**Figure 8:**
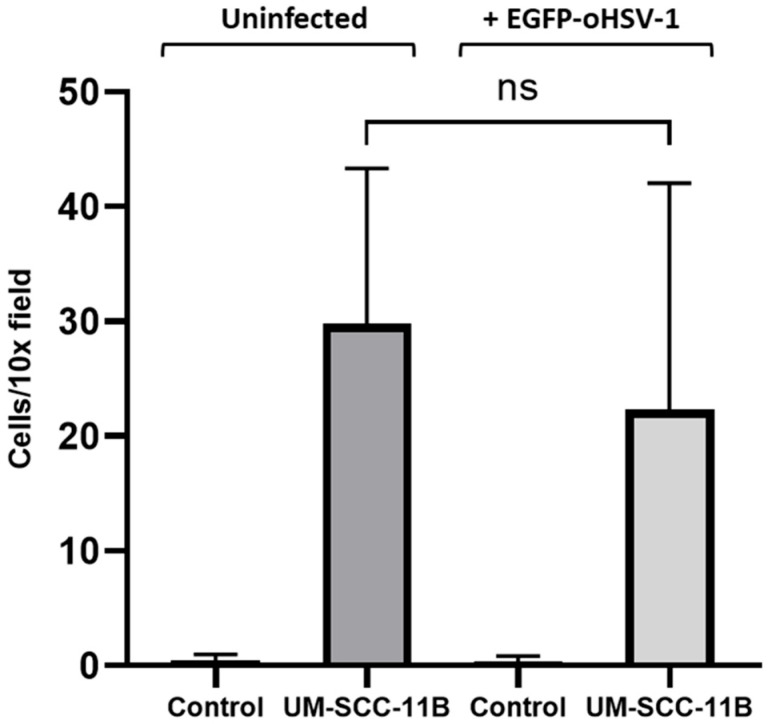
THP1 cells migrate towards serum-free medium conditioned by UM-SCC-11B cells. Uninfected or EGFP-oHSV-1 (MOI = 1 PFU/cell)-infected THP1 cells (10^5^ cells) were marked with CellTracker™ Red CMTPX Dye (Invitrogen) and allowed to migrate towards serum-free medium (Control) or serum-free medium conditioned with UM-SCC-11B cells (UM-SCC-11B) through a 5.0 µm-pore filter for 3 h at 37 °C in a 5% CO_2_ atmosphere. Fluorescent cells were counted from at least three independent 10× microscopic fields in the lower chamber. Error bars indicate standard deviation. ns stands for no statistically significant difference (*p* > 0.05, Student’s *t*-test).

Next, we demonstrated that both EGFP-oHSV-1 infected and mock-infected THP-1 cells were able to migrate towards serum-free medium conditioned by UM-SCC-11B cells, without a statistically significant difference (Figure 8).

**Figure 9:**
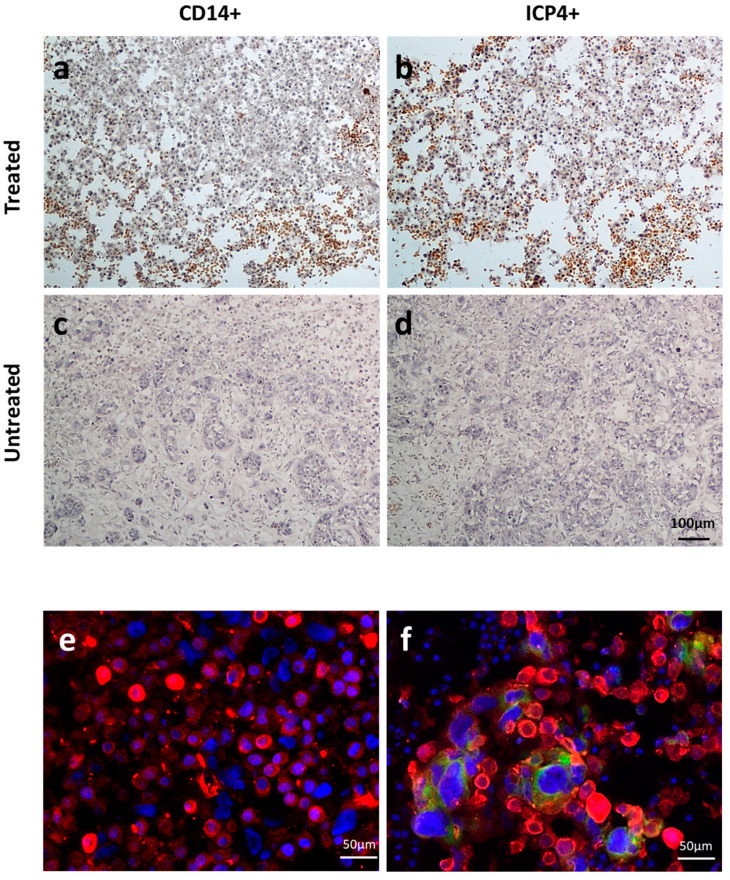
CD14^+^- and EGFP-oHSV-1-infected (ICP4^+^) cells can be observed in UM-SCC-11B-derived tumors grown on eggs injected with EGFP-oHSV-1-infected THP-1 cells. The 10× immunohistochemistry (IHC) pictures of UM-SCC-11B tumors grown on the chorioallantoic membrane (CAM) of embryonated chicken eggs. (**a**) Image of a section of an UM-SCC-11B tumor from an egg injected with intravascular EGFP-oHSV-1-infected THP-1 cells (treated) stained with primary anti-CD14 antibody and secondary horseradish peroxidase (HRP)-conjugated antibody; (**b**) image of a section of an UM-SCC-11B tumor from a treated egg stained with primary anti-ICP4 antibody and secondary horseradish peroxidase (HRP)-conjugated antibody; (**c**) 10× IHC picture of a section of an UM-SCC-11B tumor from an egg injected intravascularly with PBS (untreated), stained with primary anti-CD14 antibody and secondary horseradish peroxidase (HRP)-conjugated antibody; (**d**) 10× IHC picture of a section of an UM-SCC-11B tumor from an untreated egg, stained with primary anti-ICP4 antibody and secondary horseradish peroxidase (HRP)-conjugated antibody; (**e**,**f**) selected untreated (**e**) and treated (**f**) tumor samples were analyzed via fluorescence microscopy upon incubation with a rabbit polyclonal antibody against pan-cytokeratin wide spectrum screening (Agilent Technologies, Santa Clara, CA, USA) followed by a secondary Alexa Fluor 594-conjugated antibody (ThermoFisher Scientific, Waltham, MA, USA). Nuclei were stained with 4′,6-diamidino-2-phenylindole (DAPI, Vector Laboratories, Newark, CA, USA). Pictures were taken with an Eclipse E800 microscope equipped with DS-U1 cooled digital camera (all from Nikon Instruments, Nikon Europe B.V., Amstelveen, The Netherlands). Filters with the following characteristics were used: for EGFP excitation (EX) 480/30, dichroic mirror (DM) 505, barrier (BA) 535/45; for Alexa Fluor 594 fluorophore EX 560/40, DM 595, BA 630/60. Merged imagines are displayed. Scale bars are reported.

**Figure 10:**
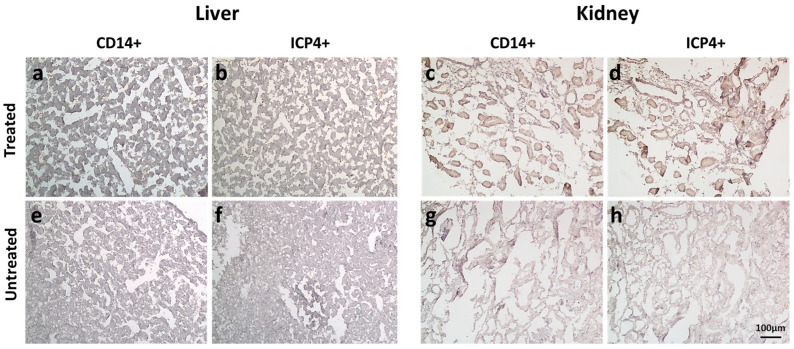
CD14^+^- and EGFP-oHSV-1-infected (ICP4^+^) cells were minimally observed in chicken embryos’ livers and kidneys following the injection of EGFP-oHSV-1-infected THP-cells. (**a**–**d**) Panels display 10× IHC pictures of representative liver (**a**,**b**) or kidney (**c**,**d**) sections obtained from EGFP-oHSV-1-injected eggs (treated) stained with primary anti-CD14 (**a**,**c**) or anti-ICP4 (**b**,**d**) antibody and secondary horseradish peroxidase (HRP)-conjugated antibody. (**e**–**h**) The same procedure was performed with control (untreated) liver (**e**,**f**) and kidney (**g**,**h**) samples. Scale bar is shown.

In a pilot experiment, THP-1 cells (5 × 10^5^ in a volume of 50 µL of PBS) or 50 µL of PBS were intravascularly (i.v.) injected into 5 embryonated chicken eggs with human UM-SCC-11B tumors growing on the chorioallantoic membrane (CAM). Data showed that monocytes can reach the tumor bed without significant effects on the embryos up to 4 days post-inoculum (Figure S3). THP-1 cells were then infected with EGFP-oHSV-1 at the MOI of 3 PFU/cell and 1 hpi were i.v. injected (5 × 10^5^ in a volume of 50 µL of PBS) in 12 embryonated chicken eggs with human UM-SCC-11B tumors growing on the chorioallantoic membrane (CAM). Twelve additional eggs bearing tumors on CAM were treated with 50 µL of PBS as a control. Since the primary goal of this experiment was to test the biodistribution of infected monocytes within the embryo tissues, one single time point post-inoculum (4 days) was taken into account. Tumors, livers and kidneys were harvested and stained using immunohistochemistry (IHC) with primary antibodies against human CD14 (a marker of monocyte carrier cells) and against the viral protein ICP4 (Figure 9a–d and Figure 10). Selected tumor sections were analyzed via immunofluorescence for the expression of EGFP and cytokeratins (marked in red), the latter ones as a tumor marker (Figure 9e,f).

**Figure 11:**
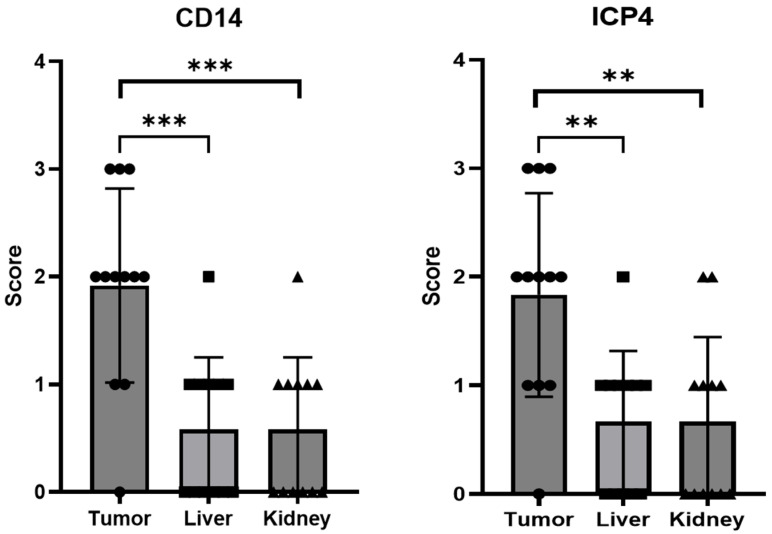
CD14^+^- and EGFP-oHSV-1-infected (ICP4^+^) cells are more frequently found in tumors than in the livers and kidneys of treated chicken embryos. Microscopy pictures of UM-SCC-11B tumors, livers and kidneys of chicken embryos were analyzed using LuciaG 5.0 software (Nikon Instruments). The difference in staging grade between tumor and liver or tumor and kidney was statistically significant (*** *p* < 0.001 and ** *p* < 0.01, Student’s *t*-test) for both CD14 (**left** panel) and ICP4 (**right** panel). Semiquantitative staging was defined as 0 = no positivity; 1 = sparse positive cells; 2 = clumps of positive cells; 3 = widespread cell positivity.

The presence of CD14- and ICP4-positive cells in the various tissue samples was evaluated semi-quantitatively by counting positive cells over multiple microscopy fields (Figure 11). This assay showed that both types of cells were present in almost all analyzed tumor sections, with the exception of one sample. Supporting these findings, four out nine analyzed tumors tested positive for oHSV-1 DNA via real-time PCR (threshold cycle range 26 to 35). On the other hand, most liver and kidney specimens tested either negative or displayed few positive cells. In agreement, when the results obtained for tumors were compared with those for livers or for kidneys, there was a statistically significant difference (Figure 11). Furthermore, the presence of infected cells within the tumors was also demonstrated using immunofluorescence assays. Indeed, in tumor sections obtained using embryos injected with infected THP1 cells, green enlarged cells were clearly visible, with some yellow areas, suggestive of infected tumor cells (Figure 9f).

## 3. Discussion

It is widely acknowledged that circulating monocytes accumulate in the microenvironment of many different tumors of epithelial [41], connective tissue [42] and hematologic origin [43], where they differentiate to tumor associated macrophages (TAMs). This finding also holds true for tumors developing in organs with an abundant population of tissue-resident macrophages, such as the central nervous system (microglia) [44] and the liver (Kupffer cells) [45]. It is also known that circulating monocytes migrate into tumors which are poorly infiltrated by T lymphocytes [29] and several research groups are trying to enhance the migration of chimeric antigen receptor (CAR) T cells to solid tumors by modifying these cells to express chemokine receptors usually found in myeloid cells [45,46].

This work provides first evidence that human monocytes derived from both a continuous cell line (THP-1 cells) and primary cells from healthy donors can function as carriers for oncolytic herpes simplex virus type 1 (oHSV-1). Indeed, we were able to show that monocytes are susceptible to infection with EGFP-oHSV-1, a virus carrying the same deletions as the clinically approved T-VEC along with a reporter gene (EGFP). However, while THP-1 cells, which are of malignant origin (monocytic leukemia), supported a productive infection, primary cells from healthy blood donors exposed to EGFP-oHSV-1 proved to be susceptible to viral infection, but were, as expected, poorly permissive. Indeed, they released few infectious particles over time and displayed a reduced percentage of EGFP positive cells, rapidly reaching 0%. In agreement, cell viability was not affected when compared to uninfected monocytes. These results confirm that the oHSV-1 we adopted here is clearly attenuated in healthy cells, as are primary monocytes compared to THP-1 cells. Furthermore, they support the notion that monocytes are poorly permissive to HSV replication [38,40,47,48]. On the other hand, and relevant for their adoption as carrier cells, we show that primary monocytes are susceptible to EGFP-oHSV-1 infection. Indeed, the cells expressed the immediate early ICP4 viral protein 24 h post-incubation with the virus, whereas they were tested negative for ICP4 at 1 h post-infection. Importantly, when infected monocytes were cultured in cell-free medium conditioned by both human breast (MDA-MB-231) and head-and-neck (UM-SCC-11B) cancer cells, the amount of infectious viral particles released as well as the number of green cells increased significantly compared to the controls. The molecular mechanisms underlying this boost in oHSV-1 replication surely deserve further studies. However, based on evidence from the literature, they could be at least partially explained by the presence of soluble factors released from cancer cells that affect the differentiation status of monocytes. Indeed, it is known that M2-polarized macrophages are more permissive to HSV-1 infection than monocytes [47] and M1-polarized macrophages [38].

We also report that infected primary monocytes were able to migrate as efficiently as mock-infected monocytes towards medium conditioned by cancer cells. Moreover, they transmitted EGFP-oHSV-1-infection to breast cancer MDA-MB-231 and larynx carcinoma UM-SCC-11B cells in coculture assays, leading over time to an almost complete infection of the tumor cell monolayer. Finally, the amounts of viral particles produced in coculture experiments were orders of magnitude higher than those obtained through infected monocytes alone, even after culturing with medium conditioned using cancer cells. This finding provides an additional indirect proof of the infection of cancer cells.

Overall, these results are very promising for the adoption of monocytes as oHSV1 carrier cells in vivo. Indeed, they suggest that monocytes could be loaded ex vivo with oHSV-1 and survive until they reach the tumor microenvironment (TME) where viral replication is boosted, with the subsequent infection of cancer cells.

To further assess the feasibility of using monocytes as carrier cells of EGFP-oHSV-1 in vivo, we employed a more complex experimental model, i.e., the chorioallantoic membrane (CAM) model. Despite the absence of a mammalian immune system, we selected this experimental setting, as it is readily accessible, easy to handle and allows the intravascular injection of therapeutics and the evaluation of the migration of infected carrier cells towards the tumor in an animal vascular system. The CAM model has already been validated as a quick and low-cost high-throughput experimental setting for testing different aspects of OV/tumor cell interactions in vivo [33,34]. In particular, the CAM system allows both an intratumoral and intravenous administration of the oncolytic agent and enables a preliminary analysis of biodistribution patterns and tumor-targeting profiles. The latter can be performed, for instance, by weighing the tumor mass upon treatment [33]. A human monocyte protein (CD14) and the immediate early viral ICP4 protein were detectable by IHC in most tested tumors, while very few ICP4- or CD14-positive cells were found in the livers and kidneys of treated chicken embryos. In addition, immunofluorescence assays highlighted the presence of EGFP-positive cells within the tumor sections, along with yellow areas suggestive of infected cancer cells, the latter being marked in red with a pan-cytokeratin antibody. It has been previously reported that HSV infection can affect cytokeratins in infected cells [49,50], a finding that might at least partially explain the observed pattern of yellow staining. Viral DNA was also detected by qPCR in four out of nine analyzed tumors, thus confirming the presence of oHSV-1 via a different method, albeit with a relatively low frequency. However, this was not unexpected, since CD14- as well as ICP4-positive cells formed clusters which could be absent in the small sections used for DNA extraction. In addition, samples were fixed in formalin, which is a known inhibitor of PCR reactions [51]. Finally, our results provide one of the first demonstrations that the CAM model is also suitable for assessing the biodistribution of cell therapy-based products.

In conclusion, while OVs have been successfully engineered to improve their selectivity and/or capacity of therapeutic gene expression, a major hurdle to the clinical translation of oncolytic virotherapy is still represented by a lack of simple, clinician- and patient-friendly delivery strategies. On one hand, certain tumors, such as melanoma, can be easily injected every two weeks, as in the case of T-VEC therapeutic schedule [13]. On the other hand, most of the deadliest human cancers are deep-seated tumors that require local treatment, which is difficult, expensive, and often painful in routine clinical practice. Carrier cells might represent a feasible alternative. However, proof-of-concept studies have not always been followed by progression towards clinical trials. One reason for this could be the difficulties in cultivating and differentiating most of the carrier cells tested so far ex vivo [18]. Moreover, even if carrier cells display a natural tropism for tumors, uptake by non-tumoral tissue cannot be excluded, thus requiring further OV manipulation, for instance, to render healthy cells more resistant to infection. The results we report here pave the way for the further testing of monocytes as a tool for systemic delivery of oHSV-1 in vivo, suggesting that the peculiar interplay between this virus and these carrier cells, with a productive infection depending on the cellular differentiation status, could favor an increased on-target cytolytic viral replication.

## 4. Materials and Methods

### 4.1. Cell Culture

Vero cells (green monkey kidney cells, ATCC CCL-81^TM^), UM-SCC-11B cells (human head and neck larynx carcinoma cells, RRID:CVCL_7716, kindly provided by Prof. Cornelia Brunner, Clinic for Oto-Rhino-Laryngology, University Medical Center Ulm), MDA-MB-231 cells (human breast cancer, ATCC HTB-26) and 293T cells (human embryonic kidney, ATCC CRL-3216) were maintained in Dulbecco’s modified Eagle medium (DMEM, Gibco, Waltham, MA, USA), supplemented with 1% *v*/*v* Penicillin–Streptomycin (Gibco) and 10% *v*/*v* fetal calf serum (FCS, Gibco). Cells grew in adhesion and were passaged twice a week and cultured at 37 °C, in a 5% CO_2_ and 98% humidity atmosphere.

THP-1 cells (human acute monocytic leukemia) were obtained from ATCC (TIB-202) and maintained in Roswell Park Memorial Institute (RPMI) 1640 medium (Gibco) supplemented with 1% *v*/*v* Penicillin–Streptomycin and 10% *v*/*v* FCS. Cells grew in suspension and were passaged twice a week and cultured at 37 °C, in a 5% CO_2_ and 98% humidity atmosphere.

Primary human monocytes were purified from the buffy coats of healthy blood donors provided by the Padua University Hospital. Briefly, peripheral blood mononuclear cells (PBMCs) were isolated from buffy coat blood via centrifugation using a Ficoll Paque Plus (Merck KGaA, Darmstadt, Germany) gradient. PBMCs were then washed in phosphate-buffered saline (PBS 1X) (Gibco) solution 4 times to remove impurities. Viable cells were counted with a hemocytometer and Trypan blue Solution 0.4% (Gibco) staining, then suspended in RPMI 1640 medium supplemented with 1% Penicillin–Streptomycin and 10% FCS at a final concentration of approximately 10^7^ cells/mL. Monocytes were purified through adhesion to a tissue culture-treated vessel. Purity was assessed using immunofluorescence staining with an anti-CD14 mouse antibody (ab181470, Abcam, Cambridge, UK) and a secondary goat anti-mouse antibody conjugated to Alexa Fluor 488 (ab150117, Abcam).

CD14-positive cells were roughly 80% of all adherent cells and were maintained as adherent cells in RPMI medium supplemented with 10% *v*/*v* FCS and 1% *v*/*v* Penicillin–Streptomycin.

The conditioned medium in which primary monocytes were cultured in some experiments was produced by culturing sub-confluents MDA-MB-231 and UM-SCC-11B in RPMI 1640 medium supplemented with 1% Penicillin–Streptomycin and 10% FCS for 24 h.

All employed cell lines were regularly tested for mycoplasma contamination by end-point PCR using the AmpliTaq Gold™ DNA polymerase (Applied Biosystems, Waltham, MA, USA), a forward 5′-GGGAGCAAACAGGATTAGATACCCT primer and a reverse 5′-TGCACCATCTGTCACTCTGTTAACCTC primer.

### 4.2. Migration Assays

In vitro migration assays were carried out using 6.5 mm Transwell plates with 5.0 μm pore polycarbonate membrane inserts (Corning, Torino, Italy). Uninfected and infected THP-1 cells and primary human monocytes (in both cases 10^5^ cells) were suspended in 100 µL serum-free medium (OptiMEM^TM^, Gibco) and seeded in the inserts, while 600 µL of serum-free medium alone or serum-free medium in which cancer cells had been cultured were placed in the well. Transwell plates were incubated for 3 h at 37 °C in a 5% CO_2_ atmosphere with 98% humidity. Inserts were removed and migrated cells were stained with CellTracker™ Red CMTPX Dye (Invitrogen) following the manufacturer’s instructions and were counted over at least three different 10× microscopy fields.

### 4.3. Viruses

Δγ34.5/ΔUs12/EGFP-oHSV-1 (hereafter named EGFP-oHSV-1) was obtained via bacterial artificial chromosome (BAC) mutagenesis using a method which has been described previously [52]. Specifically, a BAC containing the entire genome of a strain 17^+^ HSV-1 with double gamma34.5 deletion (Δγ34.5) and the insertion of a cassette expressing the Firefly Luciferase in the UL55–UL56 intergenic region was kindly provided by Prof. Beate Sodeik (University of Hannover) [53]. BAC mutagenesis was performed to achieve the same US12 deletion described in talimogene laherparepvec [12]. Thereafter, the Firefly Luciferase-expressing cassette was replaced with a similar eukaryotic expression cassette in which the expression of the enhanced green fluorescent protein (EGFP) was driven by the immediate early cytomegalovirus (CMV) promoter/enhancer. The generated BAC was transfected in 293T cells using Lipofectamine 2000™ (Invitrogen) to reconstitute EGFP-oHSV-1. Viral stocks were prepared through amplification in green monkey kidney Vero cells and quantified using plaque titration assay on Vero cells, as previously described [54].

### 4.4. Cell Infection and Coculture Assays

THP-1 cells (1 × 10^5^) were centrifuged for 5 min at 1500× *g*, washed in PBS and then suspended in serum-free RPMI 1640 medium containing the desired amount of EGFP-oHSV-1. Cells were incubated for 1 h at 37 °C in a 5% CO_2_ atmosphere with 98% humidity, centrifuged and suspended in complete medium (RPMI supplemented with 10% FCS and 1% Penicillin–Streptomycin). Infected THP-1 cells were maintained in complete medium, as described above, and supernatants were collected for viral titration at 24, 48 and 72 hpi.

Twenty-four and 72 hpi, a viability assay was performed on THP-1 cells infected with EGFP-oHSV-1 at the multiplicity of infection (MOI) of 1 and 3 plaque-forming units (PFU)/cell by adopting the Cell Proliferation Kit I (MTT) (Roche Diagnostics, Monza, Italy) and following the manufacturer’s instructions. For comparison, the same viability assay was performed on fully susceptible Vero cells infected with EGFP-oHSV-1 at the MOI of 1.

Primary monocytes growing in adhesion on tissue culture-treated multiwell plates which were washed with PBS prior to infection using serum-free RPMI 1640 medium containing the desired amount of EGFP-oHSV-1. Cells were incubated for 1 h at 37 °C in a 5% CO_2_ atmosphere with 98% humidity and washed 3 times in PBS. The absence of infectious viral particles in the final wash was confirmed through titration on Vero cells. Next, cells were cultivated in complete medium (RPMI supplemented with 10% FCS and 1% Penicillin-Streptomycin) or in conditioned medium. Supernatants were harvested for viral titration every 24 h up to 5 days post-infection.

In coculture assays, primary monocytes were infected with EGFP-oHSV-1 at the MOI of 3 and 5 PFU/cell, respectively, for 1 h in serum-free RPMI medium, as described above. Subsequently, cells were harvested or detached with a cell scraper, washed three times in PBS, finally suspended in complete culture medium and cocultured with confluent cancer cells at a 1:1 ratio. PBS from the last washing was collected to quantify carryover virions, while supernatants were collected for viral titration at 2, 5 and 8 days post-infection (MDA-MB-231 coculture) or 2, 3 and 7 days post-infection (UM-SCC-11B coculture).

Fluorescence microscopy pictures were taken on live cells with a Nikon Ti Eclipse confocal microscope or a DM IL LED fluorescence microscope (Leica Microsystems, Buccinasco, Italy) equipped with a DFC7000T camera (Leica).

### 4.5. Immunofluorescence of Primary Monocytes

Primary human monocytes (10^5^) were seeded on sterile 10 mm round cover glasses. After infection or mock infection, cells were fixed and permeabilized with a 100% methanol solution for 5 min at −20 °C. Next, cells were stained with a mouse anti-ICP4 primary antibody (ab6514, Abcam) and a secondary goat anti-mouse TexasRed-X-conjugated antibody (Invitrogen). Cell nuclei were stained with Draq5 (Invitrogen). Finally, imaging was performed using a Nikon Ti-Eclipse confocal microscope.

### 4.6. THP-1 Cell Infection for CAM Assay

THP-1 cells (2 × 10^7^) were washed and suspended in serum-free RPMI medium containing EGFP-oHSV-1 particles (MOI 3 PFU/cell). Cells were incubated for 1 h at 37 °C and carefully mixed every 15 min. Subsequently, cells were washed three times with PBS, counted again, suspended in PBS to a density of 10^4^ cells/µL and immediately used for intravascular (i.v.) injection in chicken eggs.

### 4.7. CAM Assay

Eggs were obtained from LSL Rhein Main, Dieburg, Germany, and were processed in Germany. According to German Animal Welfare Regulations, fertilized chicken eggs are not considered as living animals as long as experiments are terminated before the hatching of the chick. Therefore, no ethical approval was required. Fertilized chicken eggs were bred as previously described [32]. For the generation of human xenograft tumors, 3 × 10^6^/egg UM-SCC-11B cells dissolved in 15 µL serum-free medium and mixed with 10 µL ice-cold Matrigel (Corning) were applied on embryonic day eight. At embryonic day 11, 5 × 10^5^/egg EGFP-oHSV-1-infected THP-1 cells dissolved in 50 µL PBS were injected intravenously. Intravenous injection was performed as previously described [32]. At embryonic day 15, chicks were sacrificed via an overdose i.v. injection of 3 mg/egg propofol (Bernd Braun Chemicals, Rottweil, Germany). Tumors, livers and kidneys were isolated, fixed overnight in 2% PFA at 4 °C, dehydrated overnight in 30% sucrose at 4 °C, subsequently embedded in Tissue Tek (Sakura Finetek, Umkirch, Germany) and stored at −80 °C.

### 4.8. Real-Time PCR Assay

Real-time PCR was performed on DNA extracted from 20 μm-thick sections of tumors grown on CAM 1093 using the DNEasy Blood&Tissue Kit (Qiagen, Venlo, The Netherlands), the TaqMan™ Universal PCR Master Mix 1094 (ThermoFisher Scientific), the UL29RealtimeFor (5′-AAGAGCCGCGTGTTGTTC) and UL29RealtimeRev (5′-GTCCGAGGAGGATGTCCA) primers and the Taqman 1095 probe UL29 (5′ FAM-CCTACCAGAAGCCCGACAAGC-3′).

### 4.9. Immunohistochemistry (IHC)

Acetone-fixed, 4-µm-thick serially cut frozen tissue sections of tumor, liver and kidney (n = 12 each) were immunostained with mouse monoclonal antibodies against CD14 (Abcam ab181470) and ICP4 (Abcam ab6514). Briefly, after a 10 min incubation with Ultravision Protein Block (ThermoFisher Scientific) to block non-specific staining, samples were incubated overnight at 4 °C with primary antibodies. Then, sections were washed with phosphate-buffered saline (PBS) and incubated for 30 min with the proper secondary horseradish peroxidase-labeled anti-mouse antibody (EnVision, Agilent Technologies, Santa Clara, CA, USA) at room temperature. Following rinsing with PBS, immunohistochemical reactions were developed for 5 min using 3,3-diaminobenzedine tetra-hydrochloride (DAB, Abcam) and counterstained with Gill’s Hematoxylin no. 2 (Sigma-Aldrich). Slides were then dehydrated in pure ethanol and xylene (both Carlo Erba Reagents, Cornaredo, Italy) and mounted with EuKitt (Bio-Optica, Milan, Italy). Micrographs were collected using Eclipse E800 microscope equipped with a cooled DS-U1 digital camera and then stored and analyzed using LuciaG 5.0 software (all from Nikon Instruments, Nikon Europe B.V., Amstelveen, The Netherlands). Then, the staging of samples was performed, classifying the different samples as: 0 = no positivity; 1 = sparse positive cells; 2 = clumps of positive cells; 3 = widespread cell positivity.

### 4.10. Immunofluorescence Staining of Tumor Section

Selected tumor sections, fixed and cut as above described, were immunostained with a mouse monoclonal antibody against CD14 (Abcam, ab181470) and/or a rabbit polyclonal antibody against pan-cytokeratin wide spectrum screening (Agilent, Z0622) as a tumor cell marker. Briefly, after 10 min of incubation with Ultravision Protein Block, samples were incubated overnight at 4 °C with a mixture of primary antibodies. Then, after washing with PBS, slides were incubated for 45 min with the respective secondary Alexa Fluor 488- or 594-conjugated antibody (Life Technologies) raised in donkey and mounted with Vectashield Mounting Medium, to avoid photobleaching, and supplemented with 4′,6-diamidino-2-phenylindole (DAPI, Vector Laboratories) as a nuclear marker. Images were taken using an Eclipse E800 microscope equipped with a DS-U1 cooled digital camera (all from Nikon Instruments).

### 4.11. Statistical Analysis

Data elaboration and statistical analysis were performed using GraphPad Prism 9.4.0 or Microsoft Office Excel 16.

## Data Availability

The data presented in this study are available on request from the corresponding authors.

## Supplementary Materials

The supporting information can be downloaded at: https://www.mdpi.com/article/10.3390/ijms24119255/s1.
