# Supplementary material for: Human Monocytes Are Suitable Carriers for the Delivery of Oncolytic Herpes Simplex Virus Type 1 In Vitro and in a Chicken Embryo Chorioallantoic Membrane Model of Cancer
Source: Int J Mol Sci. 2023 May 25;24(11):9255. doi: 10.3390/ijms24119255 (PMC10253092; doi:10.3390/ijms24119255)
Supplement: Supplementary file 1 [file ijms-24-09255-s001.zip › ijms-2417049-supplementary.pdf]

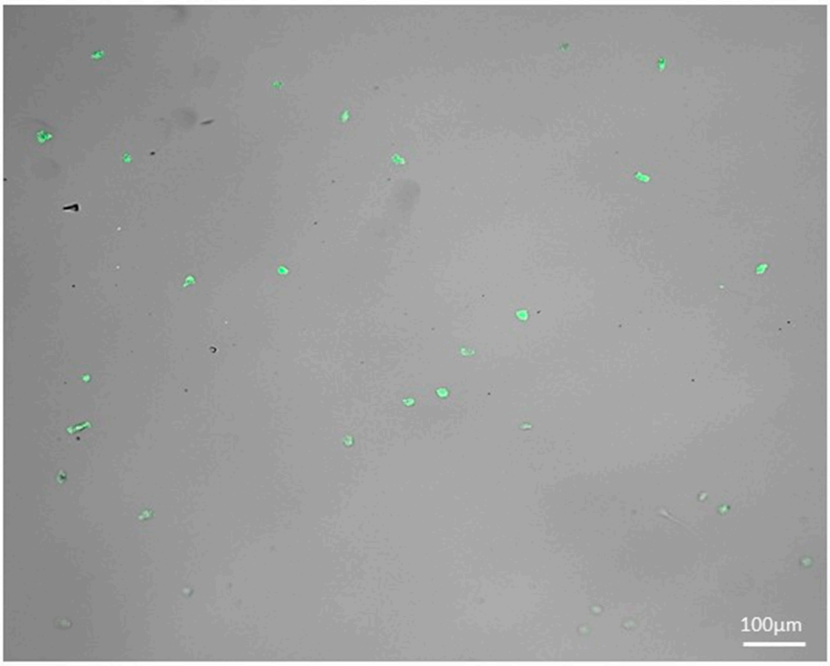

Figure S1: Enrichment of $CD14^+$ cells following purification of PBMCs. After the purification procedure described in the "Materials and Methods" section, adherent cells were fixed and permeabilized with 100% methanol, then stained with a primary mouse anti-CD14 antibody (Abcam) and a secondary goat anti-mouse FITC-conjugated antibody (Abcam).

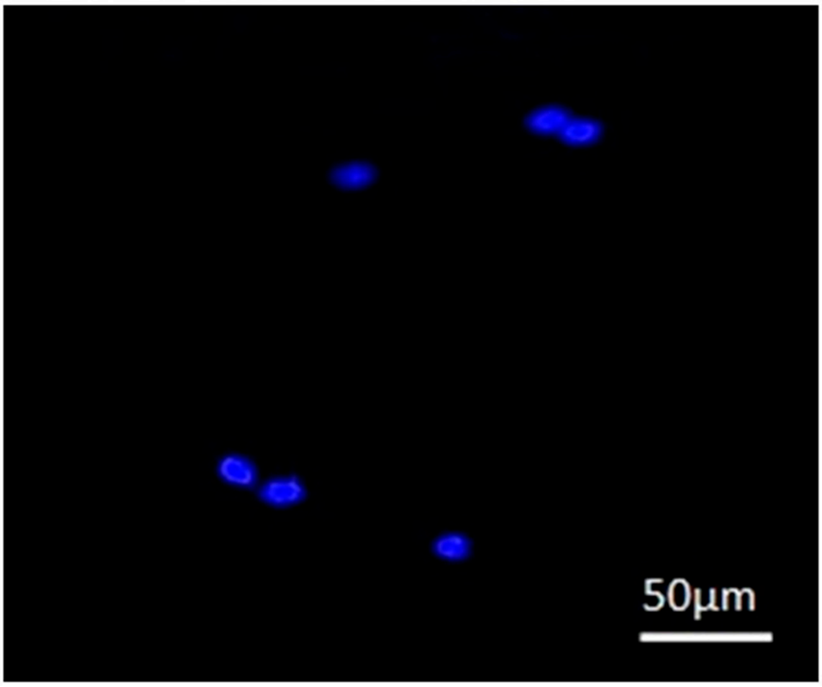

Figure S2: ICP4 positivity in infected primary monocytes is the result of de novo protein synthesis. Primary monocytes were infected with EGFP-oHSV-1 at a MOI of 5 PFU/cell. One hour after infection, reflecting viral entry but no viral protein synthesis, cells were fixed and permeabilized with 100% methanol and stained with a primary mouse anti-ICP4 antibody (Abcam) and a secondary goat anti-mouse TexasRed-conjugated antibody (Invitrogen). Nuclei were marked with Draq5 (Invitrogen). Scale bars are shown.

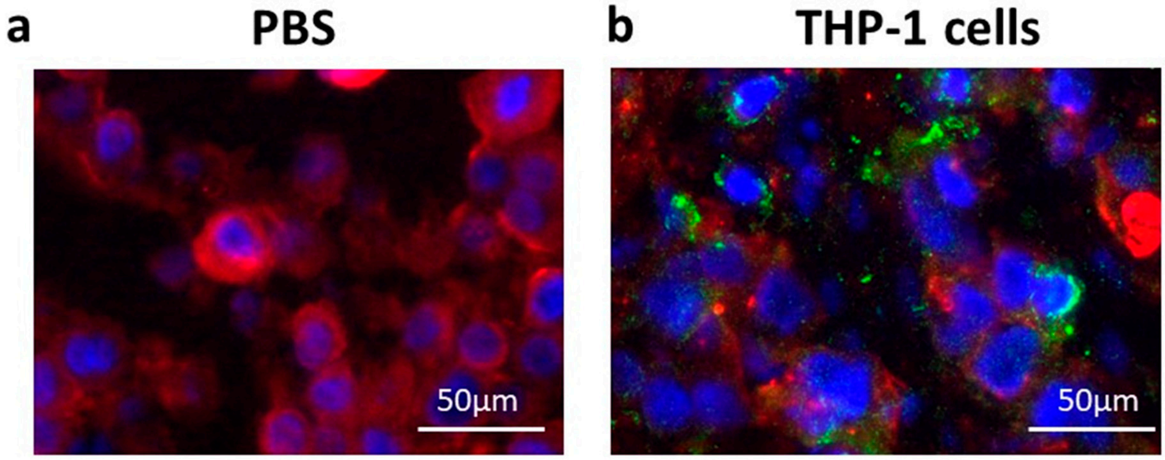

Figure S3: Uninfected monocytes migrates to tumors grown on CAM. Sections of tumor samples harvested from PBS (**a**) or uninfected THP1 cells (**b**) were analyzed via fluorescence microscopy upon incubation with a mouse monoclonal antibody against CD14 (ab181470, Abcam) and a rabbit polyclonal antibody against pan-cytokeratin wide spectrum screening (Z0622, Agilent). Next, slides were incubated with the respective secondary Alexa Fluor 488- or 594-conjugated antibody (Life Technologies) raised in donkey and mounted with Vectashield Mounting Medium, to avoid photobleaching, supplemented with 4′,6-diamidino-2-phenylindole (DAPI, Vector Laboratories) as a nuclear marker. Images were taken with Eclipse E800 microscope equipped with DS-U1 cooled digital camera (all from Nikon Instruments). Scale bars are shown.
